# Homogeneous Crystal Nucleation in Poly (butylene succinate-*ran*-butylene adipate): Challenging the Nuclei-Transfer Step in Tammann’s Method

**DOI:** 10.1021/acs.jpcb.4c06101

**Published:** 2024-11-21

**Authors:** Katalee Jariyavidyanont, Christoph Schick, Andreas Janke, René Androsch

**Affiliations:** † Interdisciplinary Center for Transfer-oriented Research in Natural Sciences (IWE TFN), Martin Luther University Halle-Wittenberg, 06099 Halle/Saale, Germany; ‡ Institute of Physics and Competence Centre CALOR, University of Rostock, 18051 Rostock, Germany; § Leibniz-Institut für Polymerforschung Dresden e.V., Hohe Street 6, 01069 Dresden, Germany

## Abstract

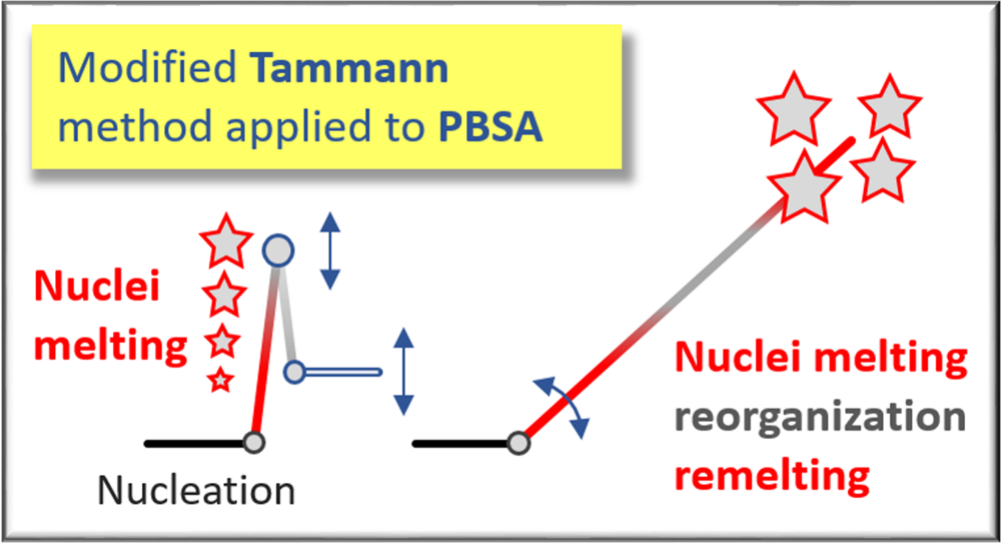

The kinetics of homogeneous crystal nucleation and the stability of nuclei were analyzed for a random butylene succinate/butylene adipate copolymer (PBSA), employing Tammann’s two-stage crystal nuclei development method, with a systematic variation of the condition of nuclei transfer from the nucleation to the growth stage. Nuclei formation is fastest at around 0 °C, which is about 50 K higher than the glass transition temperature and begins after only a few seconds. Due to the high nuclei number, spherulitic growth of lamellae is suppressed. In contrast, numerous μm-sized birefringent objects are detected after melt-crystallization at high supercooling, which, at the nanometer-scale, appear composed of short lamellae with a thickness of a few nanometers only. Regarding the stability of nuclei generated at −30 °C for 100 s, it was found that the largest nuclei of the size-distribution survive temperature jumps of close to 80 K above their formation temperature. The critical transfer-heating rate to suppress the reorganization of isothermally formed nuclei as well as the formation of additional nuclei during heating increases with the growth temperature at temperatures lower than the maximum of the crystallization rate. This observation highlights the importance of careful selection of the transfer-heating rate and nuclei development temperature in Tammann’s experiment for evaluation of the nucleation kinetics.

## 1 Introduction

Melt-crystallization of polymers proceeds via crystal nucleation and growth. Nucleation typically is classified as homogeneous or heterogeneous nucleation, with both controlled regarding their kinetics by the degree of supercooling of the melt, that is, the temperature difference between the equilibrium melting point and the crystallization temperature.^1,2^ At low melt-supercooling, or at rather high crystallization temperatures, heterogeneous nucleation is common, often proceeding on the surface of foreign particles or other heterogeneities and leading to spherulitic growth of lamellae due to the relatively (compared to homogeneous nucleation) low number of nuclei.^3^ In contrast, homogeneous crystal nucleation requires large supercooling of the melt due to the higher activation barrier, however, under this condition, often a much higher number of crystal nuclei forms, frequently causing growth of defective, nonlamellar, and small crystals.^4−8^

Understanding the kinetics of homogeneous crystal nucleation is crucial for optimizing the manufacture of polymer products through melt-processing methods. In these processes, the polymer melt may be subjected to rapid cooling,^9,10^ which may lead to solidification by crystallization via homogeneous nucleation at rather low temperature, or vitrification to yield a glass. It is worth noting that homogeneous crystal nuclei also form even when crystallization is suppressed by quenching the melt to below the glass transition temperature (*T*_g_).^11−13^ These homogeneous nuclei can then grow into crystals if the temperature is increased into a temperature range where the growth rate is sufficiently high, often called cold-crystallization, leading to a change in the structure and crystallinity of the polymer and eventually its properties.^14−16^

Quantitative information about the kinetics of homogeneous crystal nucleation cannot be obtained directly but is feasible by Tammann’s two-stage crystal nuclei development method.^17,18^ Typically, the temperatures of the maximum rate of homogeneous crystal nucleation on one side and of crystal growth on the other side are different,^19−22^ such that at temperatures near *T*_g_ the nucleation rate is high, and the growth rate is relatively low. In contrast, at higher temperatures, the nucleation rate is low but the growth rate is high. Tammann’s approach, therefore, implies formation of homogeneous crystal nuclei at high supercooling of the melt, followed by their development into crystals of detectable size at higher temperatures. This way, the detection of crystal nuclei is enabled, for example, by counting the number of crystals/spherulites through optical or higher resolution microscopy or by measuring the increase of the crystallinity developing in a predefined time interval. So far, besides applying this method to many other materials, this approach has been used to analyze the kinetics of homogeneous crystal nucleation in many polymers,^23,24^ such as poly (ε-caprolactone) (PCL),^24−26^ poly (l-lactic acid) (PLLA),^27,28^ isotactic polybutene-1 (iPB-1),^29,30^ poly (butylene isophthalate) (PBI),^31,32^ polyamides (PA),^33−35^ poly (ethylene terephthalate) (PET),^36^ poly (butylene terephthalate) (PBT),^37^ or polypropylene of different tacticity.^13,38^

Application of Tammann’s approach for analysis of the kinetics of homogeneous nucleation at a given temperature requires careful definition of experimental parameters, such as the cooling rate for transferring the equilibrium melt to the nucleation temperature, the rate of transfer of nuclei to the growth stage, or the temperature and time of the growth stage. At the nucleation stage of Tammann’s experiment, clusters/nuclei with a nucleation-time-dependent size distribution form, with a fraction of them larger than the temperature-controlled critical size. However, only clusters/nuclei larger than the critical size corresponding to the growth/development temperature, much larger than the critical size corresponding to the nucleation temperature, can grow to detectable crystals, as smaller nuclei “melt” on their transfer to the growth temperature. Recently, the importance of the transfer stage was highlighted for few polymers.^24,30,31,39^ These studies suggest that during the nucleation step, clusters/nuclei distributions with increasing sizes (and therefore stability) are generated with time. If a low transfer-heating rate is used, small nuclei may grow into more stable ones during the transfer from the nucleation to the growth step, and simultaneously, even new nuclei may form. At very slow heating, the initial clusters may even grow into crystals before reaching the growth step. In contrast, when an infinitely high heating rate is used, all initial nuclei smaller than the critical size at the development temperature get destroyed during the transfer to the growth stage. As such, in order to investigate the cluster/nuclei size distribution at the end of the nucleation stage, the transfer to the growth stage must occur at a sufficiently high transfer-heating rate to avoid any growth or reorganization of nuclei. With an inappropriately defined transfer-heating rate, the number of crystals developing in the growth stage may differ from the number of growth-temperature-controlled supercritical-size nuclei formed in the nucleation stage. Moreover, the temperature difference between the nucleation and growth step plays a further crucial role in defining the transfer-heating rate such as the larger the temperature difference, the longer is the time for the nuclei transfer, requiring even faster heating to suppress nuclei reorganization and non-isothermal nuclei formation.^24^ So far, there is only little information about the effect of the growth-stage temperature on the analysis of homogeneous nucleation.

Regarding the analysis of the stability of nuclei forming at a specific nucleation temperature, a novel strategy involving a temperature-spike on the transfer of the nuclei to the growth stage in Tammann’s development method was developed and successfully applied to PLLA and PBI.^31,39^ With variation of the spike temperature, the nuclei-size distribution is filtered to destroy all nuclei with a size lower than the critical size corresponding to the spike temperature. For both PLLA and PBI, it was found that the nuclei number gradually decreased with increasing spike temperature and that for the given nucleation conditions most of the nuclei are destroyed on heating them about 90 K above the temperature of their formation. However, due to the very limited number of studies performed so far, the maximum stability of nuclei of other polymers may differ, and there is little knowledge about the effects of the nucleation temperature and time.

The above-described open question of the combined effect of transfer-heating rate and growth-stage temperature in Tammann’s protocol is further addressed in this study, employing a butylene succinate-*ran*-butylene adipate copolymer (PBSA) as a test material. PBSA is an aliphatic biodegradable polyester with high flexibility, good mechanical performance similar to that of polyolefins, and good melt processability.^40,41^ Compared to butylene succinate homopolymer (PBS), PBSA has a lower equilibrium melting temperature (*T*_m,0_), *T*_g_, and lower crystallinity. The latter causes a lower Young’s modulus and tensile strength than that in case of PBS, however, leads to higher elongation at break, impact strength, and enzymatic degradation rate.^40−45^ With such properties, PBSA is used as sealant layer for flexible packaging, for blown film applications like bag liners, for compost bags, mulch films, agricultural films, and is an alternative biomedical material for drug delivery systems and tissue engineering.^41,46−50^ It is important noting that all properties including the biodegradability of PBSA vary with the composition of counits, that is, the ratio between butylene succinate (BS) and butylene adipate (BA) units.^42,43,45,51^ Commercial grades contain about 20–30 mol% BA units, with *T*_m,0_ and *T*_g_ being about 115 and −45 °C, respectively.^45,52,53^ There exists little information about the crystallization kinetics of PBSA.^54−60^ Crystallization of PBSA at low supercooling of the melt leads to formation of spherulites from a low number of (heterogeneous) nuclei, showing in a microscope as banded spherulites with a diameter of about 150 μm if PBSA is crystallized above 50 °C,^57,58^ while nonbanded spherulites are observed on crystallization at temperatures higher than 70 °C.^54^ Quantitative information about the kinetics of both nucleation and crystallization of PBSA and the resulting semicrystalline structures are absent, with this lack of information being a main motivation for selecting this important biopolymer in the present work. With the selection of this material, further investigation of the effect of copolymerization of the PBS homopolymer, serving as a reference, on crystallization is possible.

In summary, in this work, we aim to investigate further the importance of the individual steps of Tammann’s method, by analysis of the kinetics of homogeneous nucleation and crystallization of PBSA, including the thermal stability of such crystal nuclei. At first, analysis of non-isothermal crystallization was performed for determination of the critical cooling rates for suppressing crystallization and nucleation on cooling. Then, isothermal experiments were carried out to examine the rate of crystallization at specific temperatures. With the capability of fast scanning chip calorimetry (FSC), structures of PBSA, prepared at predefined crystallization conditions via homogeneous and heterogeneous nucleation, were investigated using polarized-light optical microscopy (POM) and atomic force microscopy (AFM). Then, the kinetics of homogeneous nucleation of PBSA at temperatures near *T*_g_ was analyzed using Tammann’s two-stage crystal nuclei development method. Nuclei prepared at a fixed temperature were subjected to different transfer-heating rates and growth-stage temperatures, which allowed us to gain information about the reorganization of nuclei and the formation of new nuclei during heating to different temperatures. With in-between heating to different maximum temperatures before growth, the thermal stability of homogeneous crystal nuclei of PBSA was analyzed, with the possibility to quantify the number of survived nuclei by observation of their density using a combination of FSC and POM.

## 2 Experimental Section

### 2.1 Material

PBSA from PTT MCC Biochem Company Limited (Bangkok, Thailand), under the trade name BioPBS FD92PM, was used in this work. It is an extrusion grade and was available in a white-pellet form. The melt-flow rate and molecular weight are 4 g/10 min (190 °C, 2.16 kg) and 130 kg/mol, respectively.^61,62^ The percentage molar concentration of BS and BA units, determined via ^1^H nuclear magnetic resonance spectroscopy, is 72 mol% BS and 28 mol% BA.^63^

### 2.2 Instrumentation

#### 2.2.1 Fast Scanning Chip Calorimetry

A power compensation Flash DSC 1 (Mettler-Toledo, Greifensee, Switzerland) equipped with an UFS 1 chip-sensor was used in the present work. The device was connected to a Huber TC100 intracooler (Peter Huber Kältemaschinenbau SE, Offenburg, Germany). The furnace environment was purged with nitrogen gas at a flow rate of 40 mL/min, and the sample-support temperature was set constant at −90 °C. Prior placing a sample onto the chip membrane, the empty sensor was conditioned and temperature-corrected according to the instrument operating instructions. Samples used for FSC were prepared from the as-received pellets using a rotary microtome CUT 5062 (Slee medical GmbH, Nieder-Olm, Germany) equipped with a tungsten-carbide knife to obtain in a first-step thin sections with a thickness of about 10 μm. The sections were subsequently reduced in their lateral size using a stereomicroscope and a scalpel to 50–100 μm and then used for analysis of the nucleation and crystallization kinetics and the thermal stability of homogeneous nuclei.

#### 2.2.2 Polarized-Light Optical Microscopy

A combination of POM and FSC techniques served for observation of microstructures of PBSA at room temperature (about 20 °C) in dependence of crystallization temperatures and to evaluate the effect of variation of spike temperatures in Tammann’s method, as in detail explained below, on nuclei survival via the density of nuclei. Samples with a larger lateral width of about 200 μm were prepared and subjected to thermal treatments using Flash DSC 1 and 2+ (Mettler-Toledo, Greifensee, Switzerland). An OPN-184 microscope (Kern & Sohn GmbH, Balingen, Germany) was employed and operated in the reflection mode using crossed polarizers. Images were captured by a DFK 33UX252 CCD camera (The imaging Source Europe GmbH, Bremen, Germany) attached to the microscope.

#### 2.2.3 Atomic Force Microscopy

The nanometer-scale structure of PBSA prepared on FSC chips via isothermal melt-crystallization at low and high temperatures, that is, at −35 and 60 °C, respectively, was investigated by AFM at room temperature (about 20 °C). A Dimension FASTSCAN AFM (Bruker, Billerica, MA, USA) operated in the peak force tapping mode was employed, using a peak-force set point of 20 mV, and silicon nitride SCANASYST-FLUID+ sensors (Bruker, Billerica, MA, USA) with a nominal spring constant of 0.7 N/m and a tip radius of 2 nm. Prior to AFM imaging, the FSC silicon nitride membranes with the samples were detached from the ceramic frame by, first, gently pressing the backside of the frame on a double-sided tape on a glass slide, followed by tapping the four corners of the membrane using a needle tip, and removing the ceramic frame to leave only the sample attached to the sensor membrane for subsequent AFM analysis.

## 3 Results and Discussion

### 3.1 Kinetics of Non-isothermal and Isothermal Melt-Crystallization

**Figure 1:**
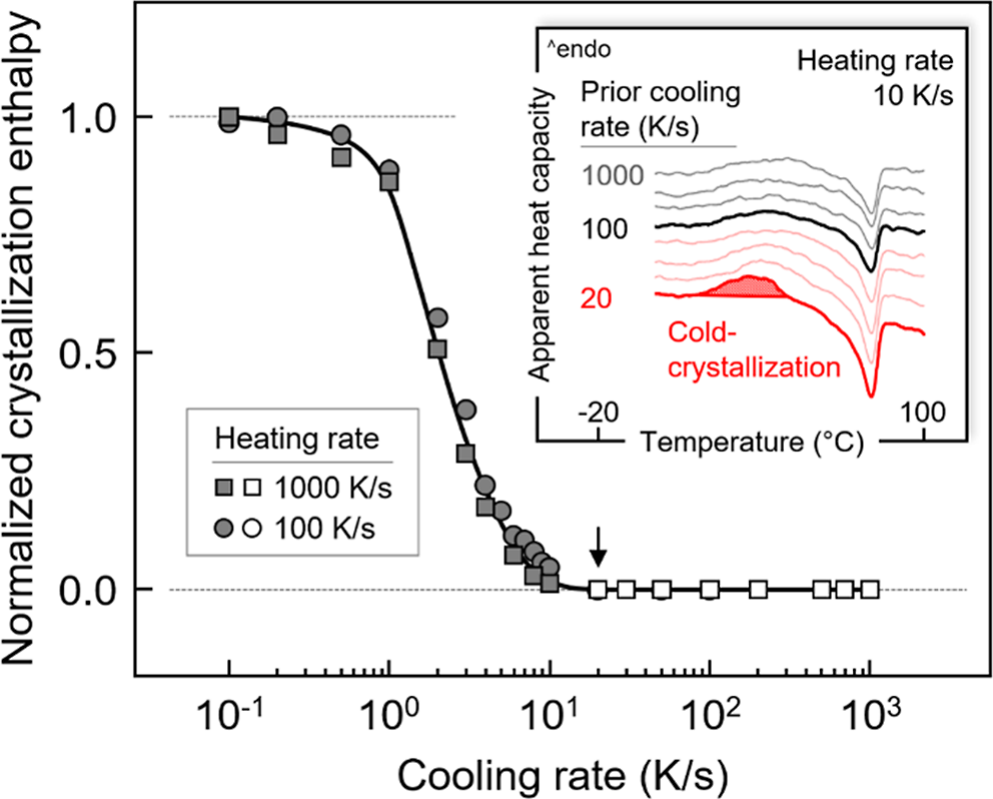
Normalized enthalpy of crystallization of PBSA as a function of cooling rate. Squares and circles represent data obtained on different samples, collected to ensure reproducibility. The inset shows the effect of cooling rate on nuclei formation, as inspected by observation of cold-crystallization on subsequent heating at 10 K/s.

Non-isothermal crystallization of PBSA was performed by cooling the relaxed melt from 120 °C to below *T*_g_, around −45 °C, using different cooling rates between 0.1 and 1000 K/s. The effect of the cooling rate on the formed crystal fraction was analyzed by subsequent measurement of the enthalpy of melting during heating at fixed rates of either 100 or 1000 K/s. The total enthalpy change during heating is equal to the crystallization enthalpy and is plotted as a function of cooling rate in Figure 1. Worth noting that enthalpies of crystallization cannot be measured during slow cooling due to the low signal-to-noise ratio of the heat-flow rate.^64^ As expected, the enthalpy of crystallization is independent of the heating rate and decreases with increasing cooling rate. With an increasing cooling rate, a slight decline of the crystallinity is detected at rates lower than about 1 K/s, and at rates between 1 and 10 K/s, the obtained crystallinity drops to zero in a narrow cooling-rate range. As such, the critical cooling rate for suppressing crystallization or crystal growth is estimated being 20 K/s (see black arrow and open data points), with this result being in agreement with the literature.^60^ However, it is important noting that even on cooling faster than 20 K/s formation of nuclei still occurs, which then causes cold-crystallization on subsequent, sufficiently slow heating, as shown in the inset.^59^ With an increase in the cooling rate, the cold-crystallization enthalpy decreases and remains constant when the cooling rate exceeds 100 K/s. Therefore, the critical cooling rate for suppressing homogeneous nuclei formation during cooling is about 100 K/s. In this work, cold-crystallization was not detected on heating faster than 100 K/s; that is, this heating rate is sufficiently fast to suppress crystal growth of nuclei formed during cooling. When compared to the PBS homopolymer, the critical cooling rates for suppressing crystallization and nucleation of PBS are about 100 and 1000 K/s, respectively, that is, about 1 order of magnitude higher than that in case of PBSA.^65,66^

In the case of analysis of the kinetics of isothermal crystallization, the relaxed melt was cooled at 1000 K/s to predefined target crystallization temperatures between −35 and 60 °C, with an interval of 5 K, and with the cooling rate selected to ensure absence of crystals and homogeneous nuclei when reaching the isothermal segment. Due to the low signal-to-noise ratio of heat-flow rate data recorded during isothermal crystallization, caused by the rather slow crystallization process, a straightforward direct determination of the half- or peak-time of crystallization is complicated. The isothermal crystallization process was therefore interrupted after predefined crystallization times, and then the fraction of formed crystals was determined by the total enthalpy change during subsequent heating at 1000 K/s.^67^

**Figure 2:**
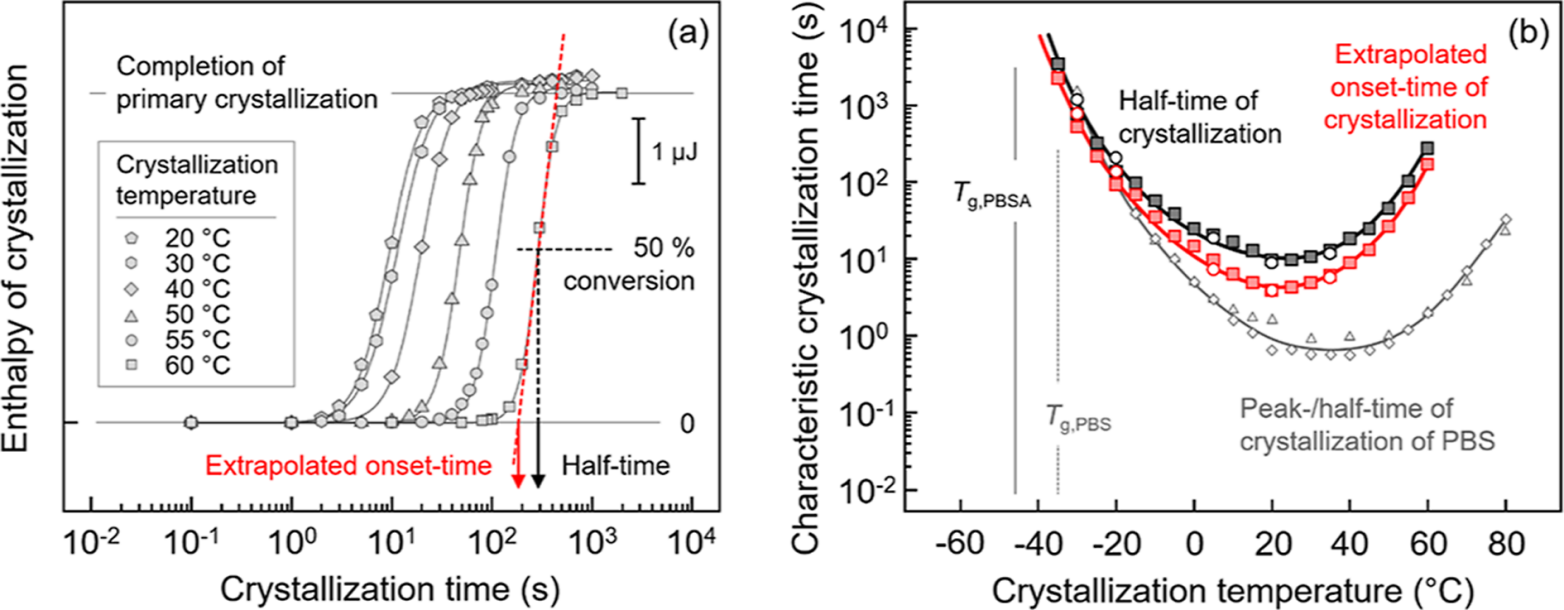
(a) Enthalpy of crystallization of PBSA at selected temperatures, as indicated in the legend, as a function of time. The arrow and dash lines in black and red colors demonstrate ways to estimate half- and extrapolated onset-times of crystallization, respectively. (b) Characteristic times of crystallization as a function of temperature. Half- and extrapolated onset-time of crystallization are presented by black and red symbols, respectively, while gray symbols represent crystallization half-times of PBS reproduced with permission from ref (66). Squares and circles represent data obtained on different samples, collected to ensure reproducibility.

Figure 2a shows the crystallization enthalpy at selected crystallization temperatures as a function of time, and Figure 2b presents characteristic times of crystallization, evaluated from the left graph as a function of temperature. Regarding Figure 2a, when the crystals grow, the enthalpy of crystallization, representing the crystallinity, rapidly increases with time until it levels off at an upper plateau. A change in the slope of the crystallization enthalpy indicates completion of primary crystallization, which is followed by a slow increase in the crystallinity due to perfection of the formed crystals via a secondary crystallization process. The half-time of crystallization is then estimated as the time needed to complete 50% of the primary crystallization process (see black line and arrow), and the begin of crystallization (see red line and arrows) is estimated by the extrapolated onset time as a more reliable measure/quantity compared to the often unsafe evaluation of the first deviation of data points from the baseline.^68^Figure 2b shows the half- and extrapolated onset-time of crystallization of PBSA, represented by black and red symbols, respectively, as a function of temperature. The reproducibility of the data is confirmed by performing measurements with two different samples, indicated by square and circle symbols. The crystallization half-time of a PBS homopolymer, available in the literature,^66^ is inserted for comparison. The temperature dependence of the characteristic crystallization-time of PBSA shows a minimum at about 20 °C, which roughly is about 70 K higher than *T*_g_. Likewise, the PBS homopolymer exhibits a similar parabolic curve of the temperature dependence of the crystallization time with a minimum at around 40 °C, about 70 K above its *T*_g_. However, the crystallization rate of PBSA is about 1 order of magnitude lower than that in the case of PBS, at least at temperatures higher than the crystallization-time minimum. Addition of a small amount of BA units into the PBS chain leads to an increase of the chain mobility (as expressed by the lowering of *T*_g_), likely due to a decrease of the density of the ester groups.^51^

### 3.2 Kinetics of Homogeneous Crystal Nucleation Using Tammann’s Two-Stage Crystal Nuclei Development Method

#### 3.2.1 Analysis of the Critical Transfer-Heating Rate to Suppress Nuclei Formation and Reorganization

**Figure 3:**
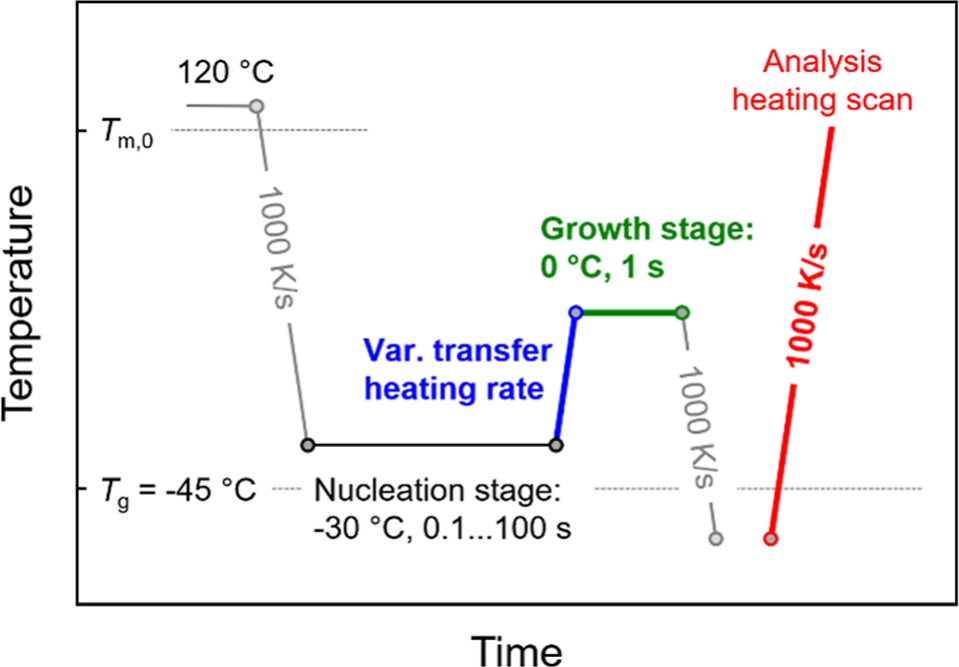
Temperature–time protocol for determination of the critical nuclei-transfer heating rate of PBSA for suppressing stabilization of nuclei within Tammann’s two-stage crystal nuclei development method.

Analysis of homogeneous crystal nucleation of PBSA was performed using Tammann’s two-stage crystal nuclei development method according to the temperature–time protocol shown in Figure 3. First, as a prerequisite to obtain reliable information about the nucleation kinetics, the critical heating rate above which reorganization/stabilization of existing nuclei and formation of new nuclei are suppressed during the transfer of the system from the nucleation to the growth stage was determined. The equilibrium melt was cooled at 1000 K/s to the nucleation temperature (black segment) of −30 °C and annealed for different times between 0.1 and 100 s. Annealing the sample at −30 °C longer than 200 s allows growth of homogeneous nuclei formed at this temperature to crystals (see red symbols at −30 °C in Figure 2b), being therefore the maximum time limit. Then, the sample was heated to the growth stage (green segment) at 0 °C, to permit crystallization for 1 s, with the growth stage approached using different heating rates between 10 and 10,000 K/s (blue segment). Finally, the crystal fraction formed in the growth stage was analyzed via the crystallization enthalpy measured during heating to 1000 K/s (red segment). Note that we do not expect continuation of both nucleation and crystallization in the cooling step between the growth stage and the analysis heating scan, due to the selected high cooling rate of 1000 K/s and rather low maximum nucleation and crystal growth rates.

**Figure 4:**
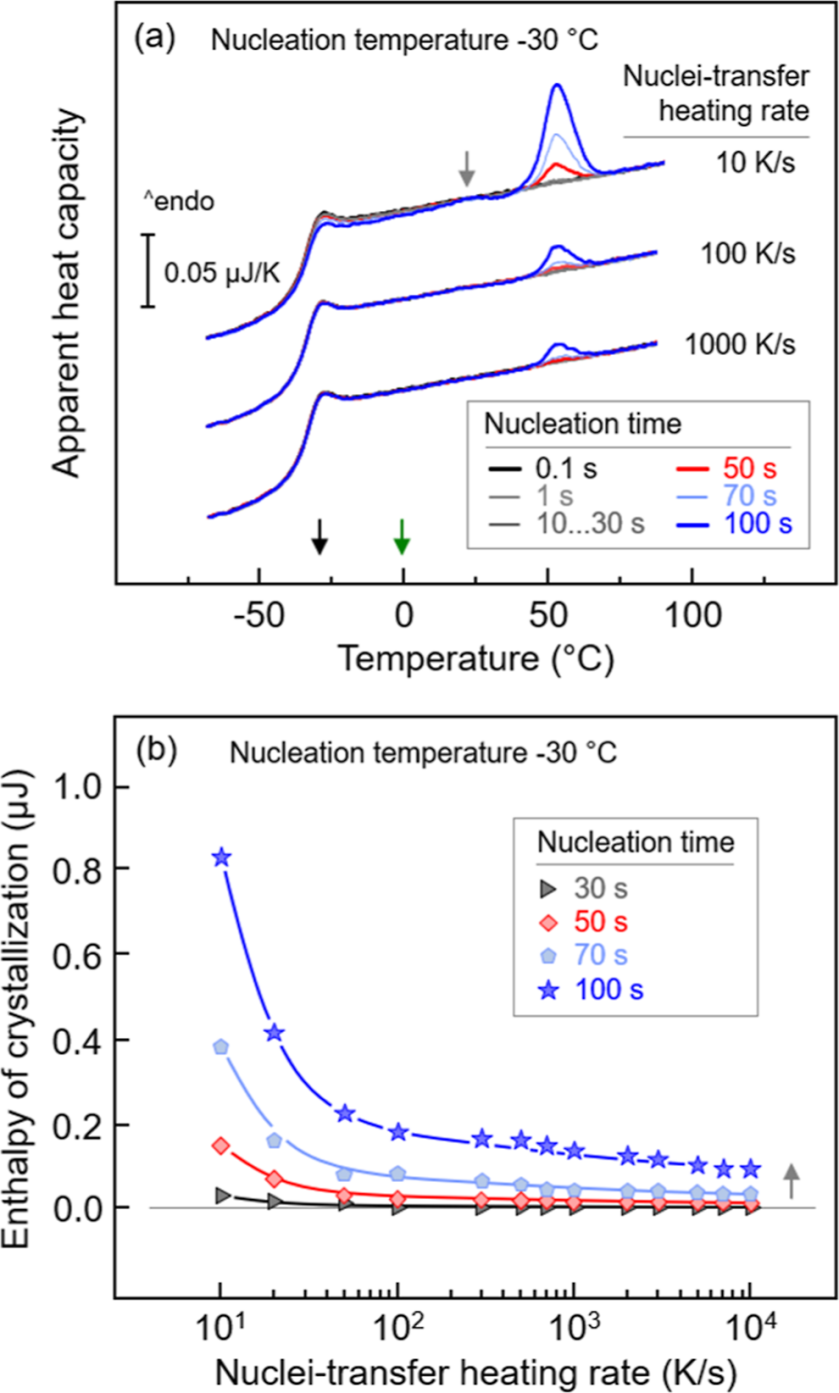
(a) Sets of PBSA heating scans collected at 1000 K/s, after transferring nuclei formed at −30 °C (black arrow) at different heating rates to the growth temperature of 0 °C (green arrow), and (b) enthalpy of crystallization as a function of the nuclei-transfer heating rate. Coloring of heating scans in (a) and symbols in (b) match each other and refer to specific nucleation times. Black and green arrows in (a) indicate the nucleation and growth temperature, respectively.

Figure 4a shows sets of analysis heating scans collected at 1000 K/s after transferring the sample from −30 °C at different rates to the growth step at 0 °C, as described in the thermal protocol shown in Figure 3, and Figure 4b shows the enthalpy of crystallization as a function of the nuclei-transfer heating rate. Coloring of both the heating scans in Figure 4a and symbols in Figure 4b corresponds to each other, representing identical nucleation time. Furthermore, the black and green arrows in Figure 4a indicate the nucleation and growth temperatures, respectively (see also Figure 3). Regarding Figure 4a, if a nuclei-transfer heating rate of 10 K/s is used (top set of curves), melting peaks in the analysis heating scan appear if the nucleation time exceeds 50 s (red curve), indicating the formation of supercritical (with respect to the growth stage) nuclei in the nucleation stage or during the transfer by further stabilization/growth of smaller nuclei. Annealing the sample at −30 °C for shorter time does not permit formation of such supercritical-size nuclei, and therefore, no melting peaks are detected (black and gray curves). When the sample is annealed at −30 °C longer than 50 s, a broad and rather small melting peak appears at around 25 °C (see gray arrow), followed by a larger peak at around 50 °C (light- and dark-blue curves). The low-temperature peak probably is related to minor crystallization already during the nucleation stage or at slow heating in the transfer stage, which, however, is not further discussed here due to the low crystallization enthalpy. If higher transfer-heating rates of 100 and 1000 K/s are used, then the melting peaks decrease in size due to a reduced number of nuclei present in the growth stage, suggesting that initially subcritical-size nuclei stabilize during their slow transfer to the growth stage. Figure 4b provides information about the effect of the nuclei-transfer heating rate on the relative (as estimated by the growth-stage crystallization enthalpy) number of homogeneous crystal nuclei at the development stage, obtained from the analysis-heating scans exemplarily shown in Figure 4a. Regardless of the nucleation time, the crystallization enthalpy strongly decreases on increasing the transfer-heating rate from 10 to 100 K/s. Then, at a higher transfer-heating rate, the enthalpy of crystallization gradually decreases further. These results demonstrate that a large number of nuclei stabilize during heating the system to the growth step if the nuclei-transfer heating rate is lower than 100 K/s, not revealing the true number of nuclei at the nucleation temperature, which exhibit supercritical size corresponding to the development/growth temperature. When a heating rate of >1000 K/s is used, stabilization of nuclei during heating is negligible. The varying plateau levels observed as a function of the nucleation time suggest differences in the nuclei-size distribution formed at −30 °C, such that with increasing nucleation time, the number of supercritical-size nuclei increases (see vertical gray arrow). Therefore, the appropriate nuclei-transfer heating rate for transferring the homogeneously formed crystal nuclei to the growth step at 0 °C is above 100–1000 K/s. Similar critical heating rates above which reorganization/stabilization of nuclei is prevented were obtained for PLLA and PET,^36,39^ however, for PBI a much lower value of 20 K/s was reported.^31^

#### 3.2.2 Analysis of the Effect of Growth Temperature on the Critical Nuclei-Transfer Heating Rate to Avoid Nuclei Stabilization

**Figure 5:**
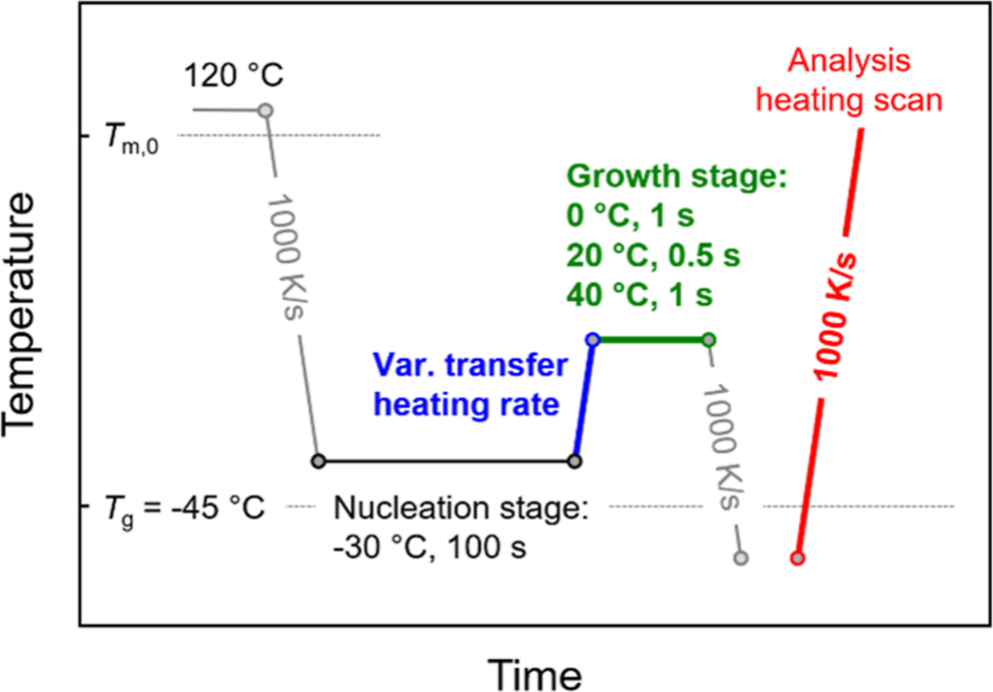
Temperature–time protocol for investigation of the effect of growth temperature on the critical nuclei-transfer heating rate of PBSA to avoid reorganization/stabilization of nuclei during their transfer to the growth stage.

Figure 5 shows the temperature–time protocol for analysis of the effect of growth temperature on the critical nuclei-transfer heating rate for preventing reorganization and stabilization of nuclei during their transfer to the growth step. First, the melt is quenched at 1000 K/s to the nucleation temperature, predefined at −30 °C, to allow nuclei formation for 100 s (black segment). Then, the sample was heated with various transfer-heating rates to different growth temperatures between 0 and 40 °C (blue and green segments). The time spent at each growth temperature was determined by the onset/begin of crystallization of the quiescent melt, not containing nuclei formed at −30 °C (see also the red symbols in Figure 2b). As such, the growth times at 0, 20, and 40 °C are 1, 0.5, and 1 s, respectively. After that, the sample was quenched to below *T*_g_ and heated at 1000 K/s for evaluation of the crystal fraction formed in the growth step (red segment).

**Figure 6:**
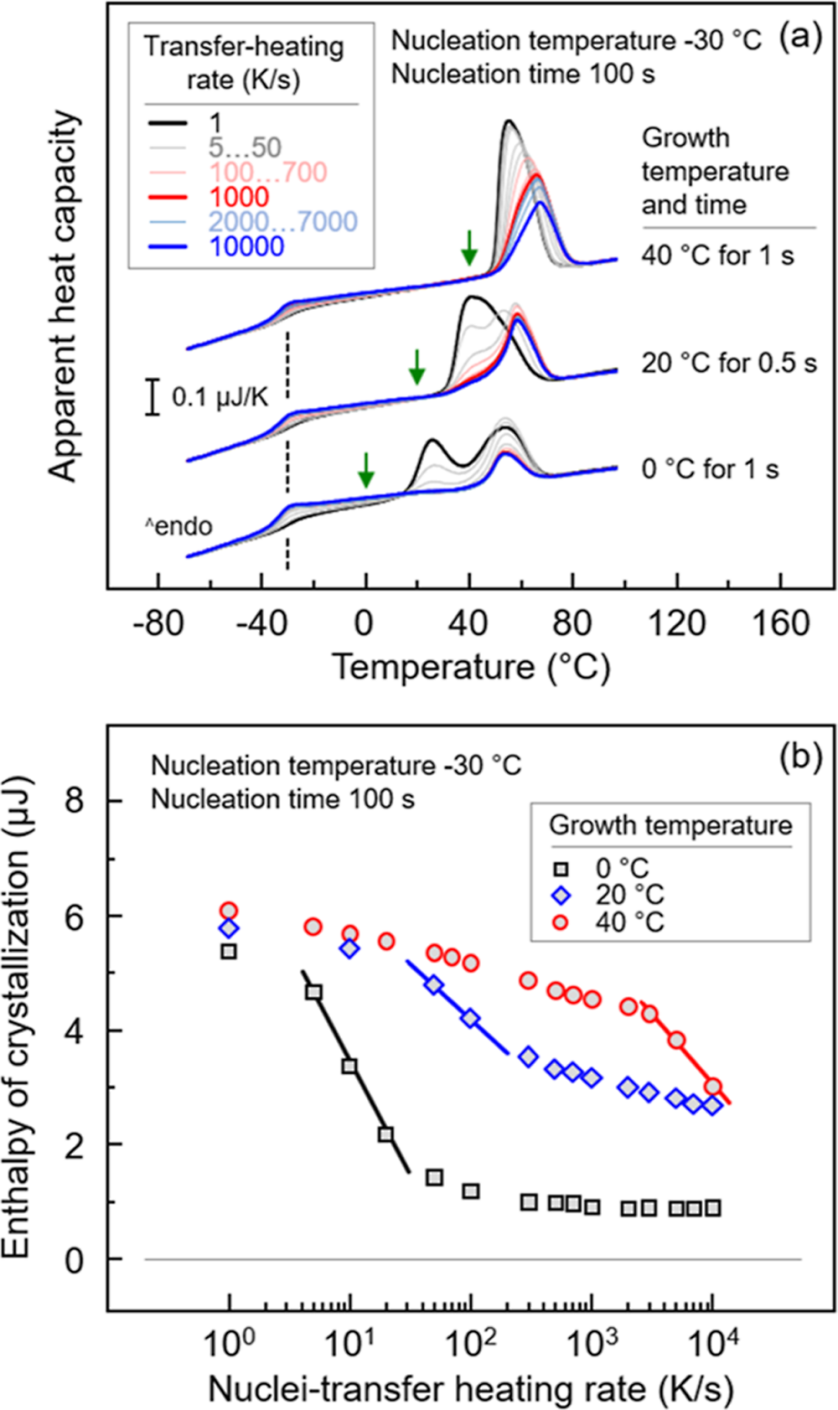
(a) Sets of PBSA heating scans collected at 1000 K/s, after transferring nuclei, formed at −30 °C for 100 s, at various heating rates to different growth temperatures between 0 and 40 °C, as indicated at each set of curves. (b) Enthalpy of crystallization of PBSA at different growth temperatures as a function of the nuclei-transfer heating rate.

In analogy to Figure 4, Figure 6a presents sets of heating scans collected at 1000 K/s after transferring the homogeneous nuclei formed at −30 °C for 100 s at various rates, as indicated in the legend, to different growth temperatures between 0 and 40 °C, while Figure 6b shows the enthalpy of crystallization as a function of nuclei-transfer heating rate. Regarding Figure 6a, the growth temperature is indicated with green arrows for each data set. The bottom set of curves refers to the growth temperature of 0 °C, with the thermal protocol used for this experiment being similar to that of the experiment depicted in Figure 4. When heating rates lower than few tens of K/s are used for transferring the sample from the nucleation stage to the growth stage (black and gray curves), distinct double melting is detected. With increasing transfer-heating rate, the low-temperature melting peak at around 25 °C becomes very small, however, still detectable. As described above, the high-temperature melting event is related to melt-recrystallization during heating crystals at 1000 K/s formed either during the nucleation stage, the transfer stage, or the growth stage, depending on the transfer-heating rate. The strong increase of the area of the melting peak at 25 °C on slow transfer of the nuclei formed at −30 °C is probably related to further growth/reorganization of crystals already present after the nucleation stage.

Likewise, the data obtained at growth temperatures of 20 and 40 °C show qualitatively similar results. At the growth temperature of 20 °C (center set of curves), the heating scans show double melting at about 40 and 60 °C. Increasing the growth temperatures allows the formation of more perfect crystals, leading to higher melting temperatures. The lower melting peak decreases in size with increasing transfer-heating rate, indicating suppression of the reorganization/stabilization of nuclei/crystals during the transfer step. When transfer-heating rates are higher than 1000 K/s (red curve), the area of both melting peaks becomes constant. With an increase in the growth temperature to 40 °C, distinct double melting is not detected, presumably due to superimposing of the two melting events. In addition, the melting peak continuously decreases in size with the transfer-heating rate up to 10,000 K/s.

The crystal fraction, as quantified by the enthalpy of crystallization, formed at growth temperatures of 0, 20, and 40 °C, is shown with black, blue, and red symbols, respectively, in Figure 6b. At the lowest transfer-heating rate of 1 K/s, the crystallization enthalpy of the samples obtained at the various growth temperatures is similar, suggesting that a similarly large number of nuclei was present at the beginning of the growth stage. With increasing transfer-heating rate, a drastic decrease of the crystallization enthalpy in the case of the growth-stage temperature of 0 °C is observed, becoming constant at transfer-heating rates higher than about 100 K/s, being in agreement with the data of Figure 4. This decrease is interpreted as a critical heating rate above which there is no reorganization/growth of subcritical nuclei nor formation of nuclei during heating the system from the nucleation to the growth temperature. Surprisingly, the critical heating rate increases with increasing growth temperature of 20 and 40 °C to 100 and above 10,000 K/s, respectively, as is indicated with the estimated highest-slope lines in Figure 6b. Obviously, the reorganization and stabilization of nuclei depend on the growth temperature. The different plateau level/crystallinity at the highest transfer-heating rates probably is caused by different crystal growth rates. Further information on nuclei reorganization/stabilization is available in Section 3.4, with Figure 11.

#### 3.2.3 Temperature Dependence of Homogeneous Crystal Nucleation

**Figure 7:**
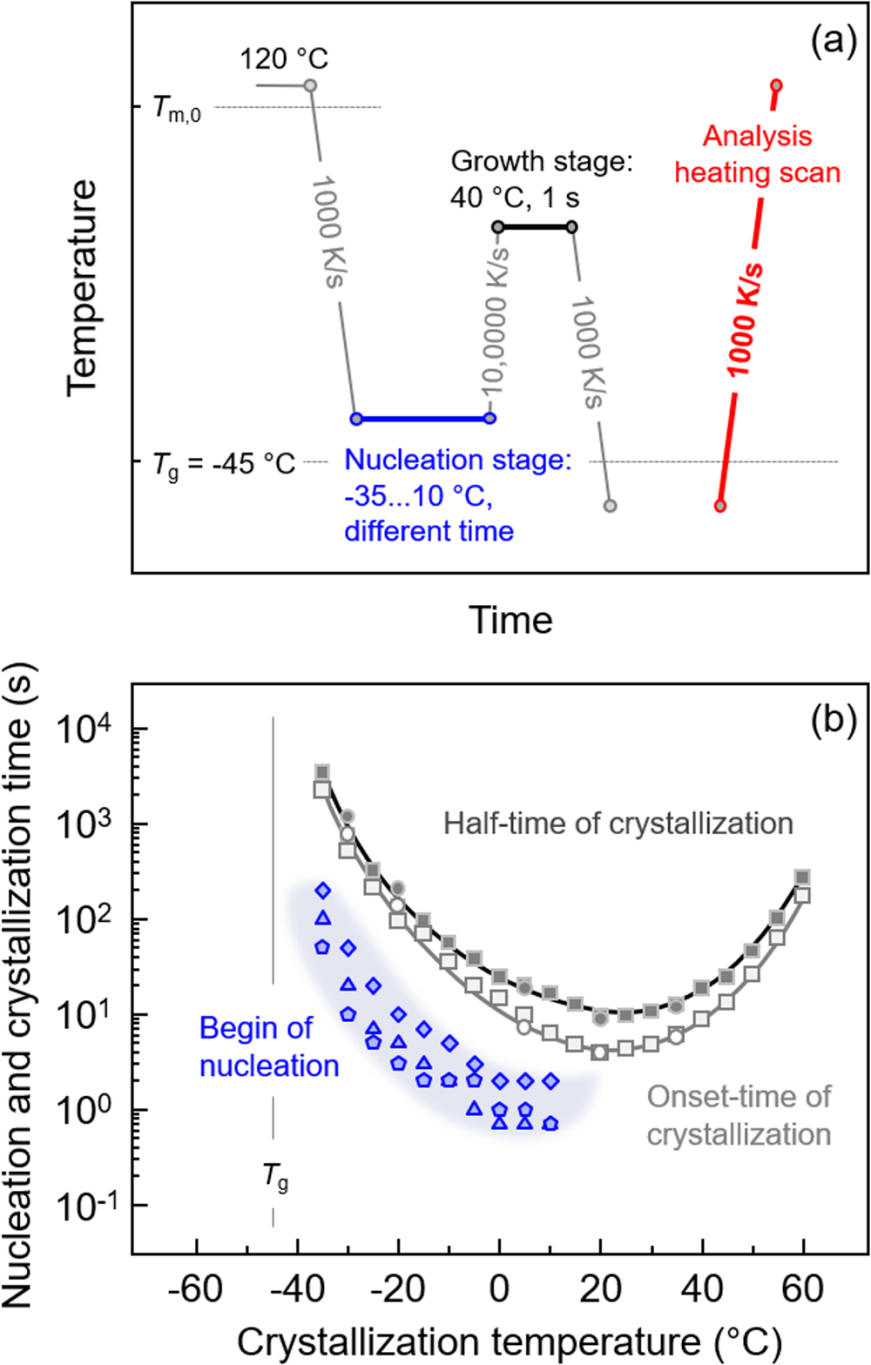
(a) Temperature–time protocol for investigation of the kinetics of homogeneous crystal nucleation using Tammann’s two-stage crystal nuclei development method and (b) characteristic time of nucleation (blue symbols) and crystallization (gray) of PBSA as a function of temperature. Various symbols represent data obtained on different samples, collected to ensure reproducibility.

Analysis of the temperature dependence of the characteristic time of homogeneous crystal nucleation of PBSA was performed using Tammann’s two-stage crystal nuclei development method according to the temperature–time protocol shown in Figure 7a. Figure 7b presents the characteristic times of nucleation and crystallization as a function of temperature. As such, the melt was quenched at 1000 K/s from 120 °C to nucleation temperatures ranging from −35 and 10 °C (blue segment), where the samples were annealed for different times, depending on the nucleation temperature. In detail, the maximum annealing time was defined by the onset time of crystallization (see also the red symbols in Figure 2b). Then, the sample was heated at a supercritical rate of 10,000 K/s to the growth temperature which was set at 40 °C, lasting 1 s (black segment), allowing the growth of crystals from homogeneous nuclei, which survived the transfer to the growth stage without reorganization. Note (a) that the growth time of 1 s is short enough to avoid formation of additional nuclei at the growth step but is sufficiently long to allow crystal formation in case of presence of nuclei, and (b) that the rather high growth stage temperature allows analyzing a broad range of nucleation temperatures. Then, the sample was quenched to below *T*_g_ before the final analysis heating scan was recorded at 1000 K/s (red segment). If the time of annealing the sample at the nucleation temperature is too short, then there is no formation of additional, with respect to permanently present heterogeneous nuclei, which would cause formation of a detectable fraction of crystals within 1 s at the growth temperature. However, when the sample is annealed long enough, allowing generation of nuclei, those nuclei grow to crystals, with their fraction analyzed by the enthalpy of crystallization in the analysis heating scan. As the number of nuclei increases with the nucleation/annealing time, the crystallization enthalpy increases correspondingly, and from plots of the enthalpy of crystallization as a function of the nucleation time, the beginning of nucleation can be determined, as shown with the blue symbols as a function of temperature in Figure 7b for three different samples, to demonstrate the reproducibility. The characteristic times of crystallization are also shown in that plot for comparison (gray symbols). The data reveal that homogeneous nucleation begins at an about 1 order of magnitude shorter time than the onset-time of crystallization, with the highest nucleation rate detected at 0–10 °C, which is about 50 K above *T*_g_.

### 3.3 Semicrystalline Structures Forming at Low and High Supercooling of the Melt

**Figure 8:**
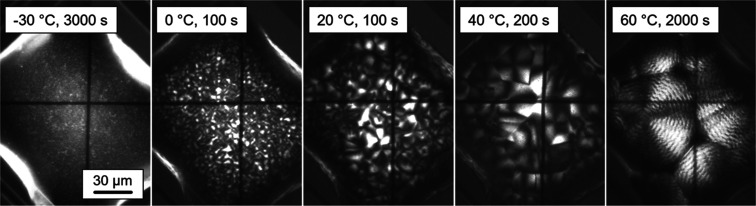
POM images of PBSA prepared by FSC by crystallization at different temperatures and for different time, indicated in each image. The scale bar holds for all images.

Figure 8 presents POM images of PBSA prepared on the FSC chip-sensor by isothermal melt-crystallization at different temperatures between −30 and 60 °C for different times, selected to complete primary crystallization, as indicated in each image. Crystallization near *T*_g_, at −30 °C, via homogeneous nucleation, leads to the formation of tiny birefringent objects with a size smaller than or around 1 μm, similar as in case of PBS.^66^ Such structure, however, differs from that of other polymers like isotactic polypropylene (iPP), PA, or PBT which revealed absence of any micrometer-scale features in POM, probably due to the large number of nuclei.^69−73^ Nucleation near *T*_g_ commonly results in extremely high nuclei densities of the order of 10^24^ m^–3^,^74^ and the formation of birefringent objects requires the coalescence of the nanometer-sized crystals growing from each single nucleus. When PBSA is crystallized at higher temperatures of 0 and 20 °C, spherulites with a diameter of about 10 μm are observed, indicating either coalescence into larger objects or a lower number of homogeneous nuclei compared to the above listed examples. On increasing the crystallization temperature further to 40 and 60 °C, even larger spherulites are detected, showing distinct banding being in agreement with independent studies,^57,58^ and probably representing the low number of heterogeneous nuclei. The distinctly finer structure of PBSA crystallized at −30 °C, compared to crystallization at 0 °C, probably is caused by the much longer nucleation time of close to 1000 s at −30 °C, compared to about only 10 s at 0 °C, before the start of crystallization, regardless of the nucleation rate, being expected higher at 0 °C (see Figure 7b).

**Figure 9:**
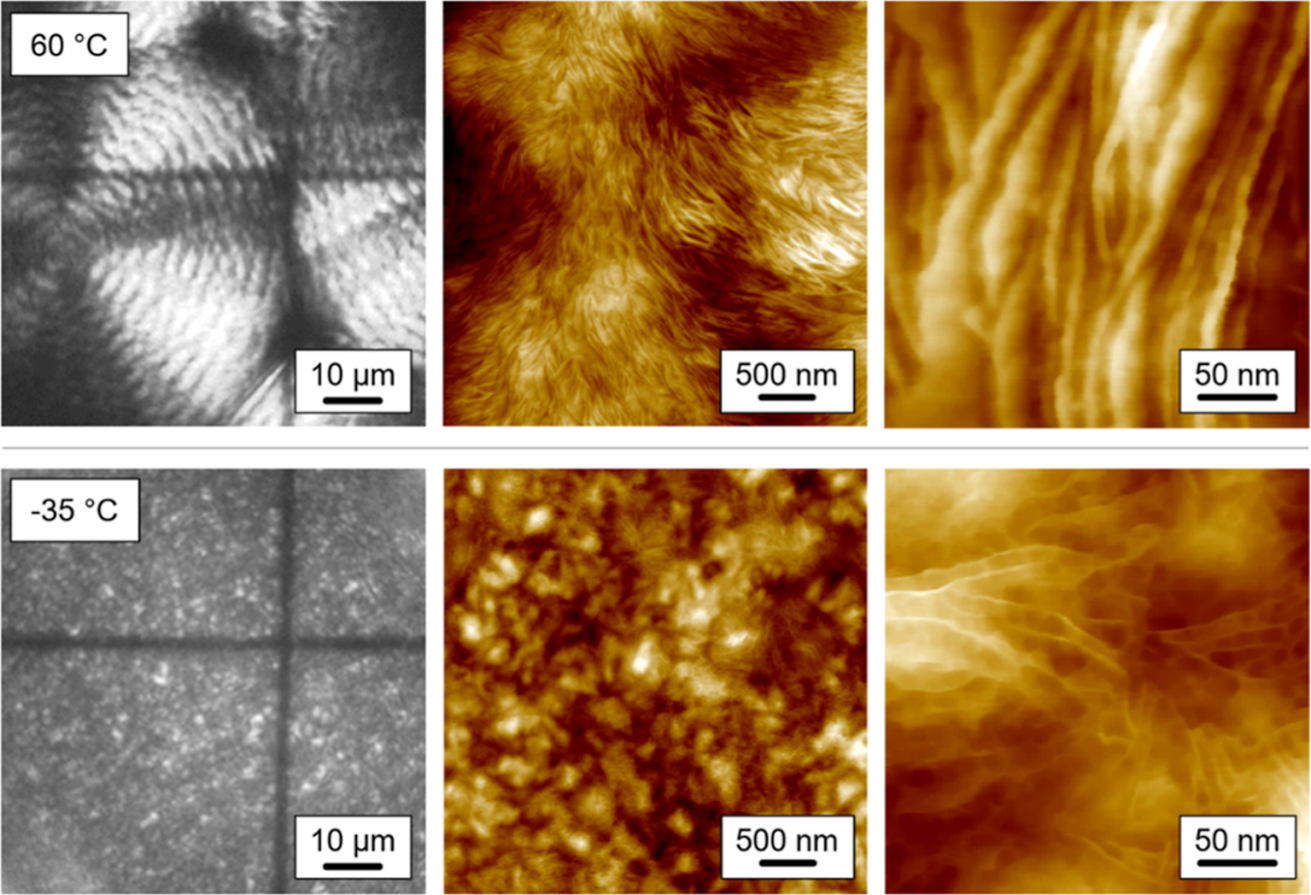
POM (gray, left column) and AFM images (colored, center and right columns) of PBSA prepared by isothermal melt-crystallization at 60 (top row) and −35 °C (bottom row).

To shed further light onto the nanometer-scale fine-structure of birefringent objects, Figure 9 shows POM images (grayscale, left column) and AFM height-images (colored, center, and right columns) of FSC samples prepared by isothermal melt-crystallization at 60 and −35 °C, presented as sets of images at the top and bottom rows, respectively. Banded spherulites grown at high temperature of 60 °C contain long lamellae with a thickness less than about 10 nm (top row). Note that the detected thickness dimension of edge-on viewed lamellae in the AFM analysis is larger than the actual size due to so-called tip-smearing,^75,76^ and that tilt of lamellae may introduce a further error when attempting to quantify the true thickness dimension. However, the obtained result is consistent with other studies, presenting the microstructure of banded spherulites with detection of long periods in stacks of lamellae of 7.9 and 8.2 nm measured by small-angle X-ray scattering when PBSA is isothermally crystallized at 52 and 66 °C, respectively, suggesting lamellar thicknesses of 3–5 nm if the linear crystallinity is around 50%,^57,58^ similar thin as in case of the PBS homopolymer.^77^ Presence of a rather low number of heterogeneous crystal nuclei typically yields formation of such lamellae and spherulites when crystallization takes place at low supercooling of the melt (top row).^78,79^ In contrast, crystallization of PBSA at −35 °C, similar to the sample crystallized at −30 °C, does not allow the formation of spherulites but displays tiny birefringent objects in the micrograph (bottom row, left). The AFM image reveals the presence of numerous sub-μm-sized domains (bottom row, center) and even isolated, short, and thin lamellae at higher resolution (bottom row, right). Qualitatively similar structure showing such domains formed during crystallization at high supercooling of the melt has also been observed for PBS.^66^ Recently, also for PLLA coalescence of tiny crystals grown from homogeneous nuclei to yield larger birefringent structures was suggested.^80^ Preparation, storage, and investigation of the samples at room temperature, which is about 50 K above *T*_g_, may enhance such a process.

### 3.4 Thermal Stability of Homogeneous Crystal Nuclei Estimated Using a Spike-Modified Tammann’s Two-Stage Crystal Nuclei Development Method

**Figure 10:**
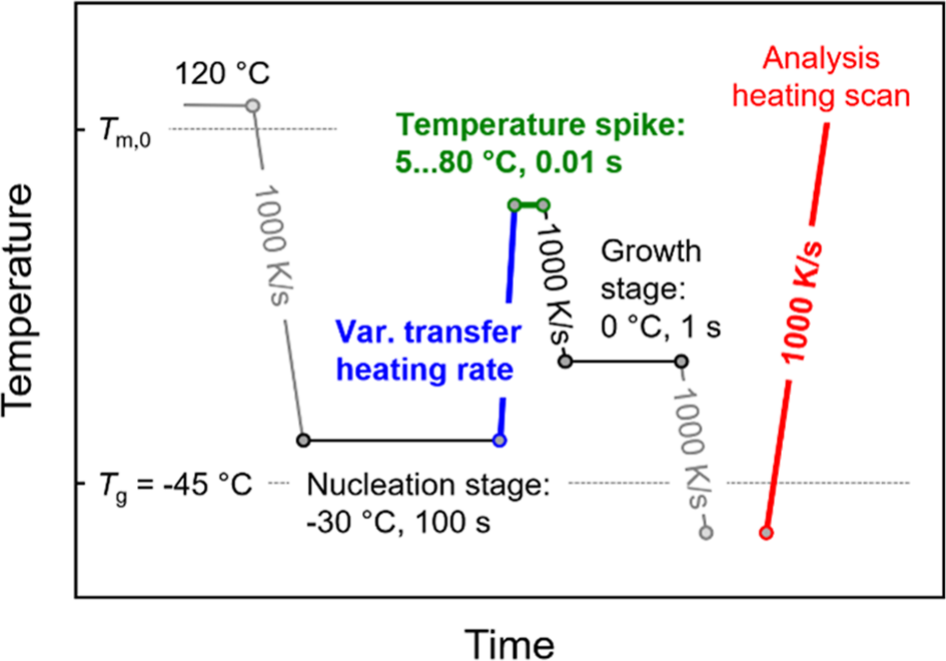
Temperature–time protocol for analysis of the thermal stability of homogeneous crystal nuclei of PBSA using a spike-modified Tammann’s two-stage crystal nuclei development method.

Figure 10 shows the temperature–time protocol for analysis of the thermal stability of homogeneous crystal nuclei using a temperature-spike-modified Tammann’s two-stage crystal nuclei development method. The nucleation and growth temperatures are −30 and 0 °C, respectively, with the corresponding annealing times at these temperatures being 100 and 1 s, respectively. The transfer-heating rate was varied from 2000 to 10,000 K/s (blue segment), preventing in all cases reorganization and stabilization of nuclei (see Figure 4b and black squares in Figure 6b). Different spike temperatures from as low as 5 °C—possible due to the selection of a low growth-stage temperature—to 80 °C were used, varied with an interval of 5 K, while the spike time was kept constant at 0.01 s (green segment). Then, the sample was quenched to the growth step at 1000 K/s to prevent both non-isothermal crystallization or formation of additional nuclei before reaching the growth temperature. After the growth stage, the crystallinity developed within 1 s at 0 °C was analyzed with the final analysis heating scan using a rate of 1000 K/s (red segment).

**Figure 11:**
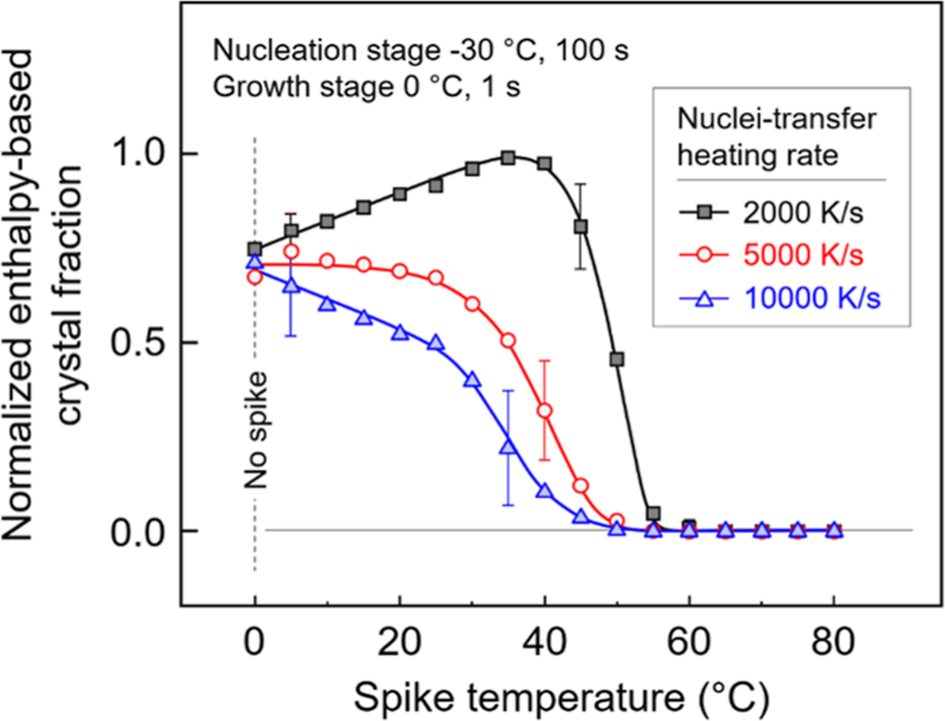
Normalized enthalpy-based crystallinity of PBSA developed at the growth stage as a function of spike temperature. The nucleation step was fixed at −30 °C, lasting for 100 s, while the growth temperature was 0 °C, allowing growth for 1 s (see also vertical dash line). Various nuclei-transfer heating rates of 2000, 5000, and 10,000 K/s were employed, indicated by black, red, and blue coloring of data, respectively.

Figure 11 illustrates the normalized enthalpy-based crystal fraction formed at the growth stage as a function of the spike temperature. The data were normalized by dividing all values by the obtained maximum crystal fraction of the data set associated with a nuclei-transfer heating rate of 2000 K/s. The vertical dash-line indicates the growth temperature and absence of an in-between temperature spike on the approach of the growth step. Various nuclei-transfer heating rates of 2000, 5000, and 10,000 K/s were employed, as indicated in the legend, being all higher than the critical heating rate for preventing reorganization and stabilization of nuclei during direct heating to the growth temperature including assuring absence of crystal formation before the beginning of the growth stage. Error bars are shown for selected conditions, serving as a demonstration of reproducibility. When a transfer-heating rate of 2000 K/s is used (black symbols), the enthalpy-based crystallinity (which scales with the nuclei number) is increasing with the spike temperature, up to 40 °C, corresponding roughly to the temperature of maximum crystal growth rate (see Figure 2b). This observation suggests the growth of crystals/nuclei during the transfer step, above the growth temperature. The obtained data are consistent with the results of Figure 6b, which showed an increase of the crystallization enthalpy when increasing the growth temperature from 0 to 40 °C at the transfer-heating rate of 2000 K/s. It should be noted that dissolution of less stable nuclei formed during the nucleation step may also occur during heating at 2000 K/s between 0 and 40 °C. When heating the sample to a spike temperature higher than 40 °C, the enthalpy of crystallization decreases until approaching zero at a spike temperature of 55 °C. This decrease demonstrates dissolution of nuclei, starting with the least stable/smallest sizes of nuclei at 45 °C until the most stable ones are destroyed at 55 °C. When a transfer-heating rate of 5000 K/s is applied, the enthalpy-based crystallinity first, at low spike-temperatures, remains constant up to 20–25 °C and then decreases with increasing spike temperature. The plateau at low spike-temperatures indicates that dissolution of nuclei during the transfer step does not significantly reduce the number of nuclei growing at the development temperature because of a possible stabilization/growth of undercritical nuclei at this heating rate. At higher spike temperatures, more and larger nuclei are destroyed, drastically reducing the number of growing nuclei. Finally, all crystal nuclei vanished at 50 °C, and the crystallinity developed in the growth stage is zero. In the case of using a nuclei-transfer heating rate of 10,000 K/s, effectively preventing stabilization/growth of nuclei, a slight but continuous decline of the crystallinity is detected up to around 25 °C, before the crystallinity then significantly decreases when the spike temperature exceeds 25 °C, reaching zero at about 50 °C, being identical to the data obtained with the transfer-heating rate at 5000 K/s. The increase of the nuclei number at low spike temperatures, e.g., at 20 °C, on decreasing the transfer-heating rate is caused by reorganization and stabilization of nuclei, being consistent with the findings presented in Figure 6.

**Figure 12:**
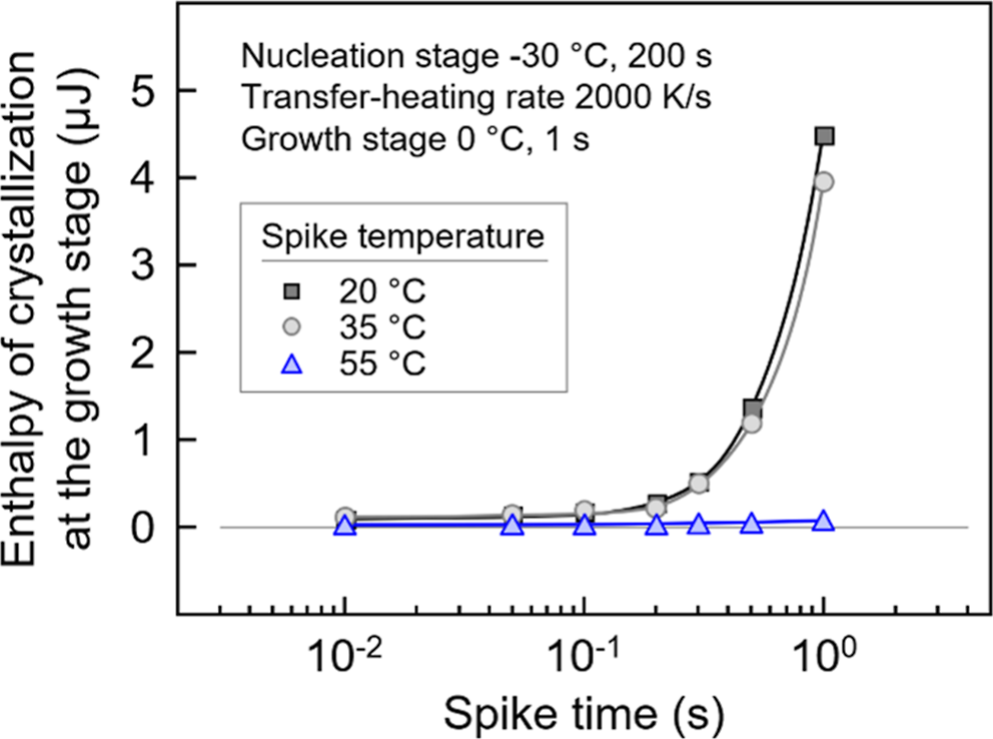
Enthalpy of crystallization/crystal fraction of PBSA formed at the growth stage at 0 °C as a function of spike time in the spike-modified Tammann’s two-stage crystal nuclei development method. The nucleation and the transfer-heating rate are fixed to −30 °C for 200 s and 2000 K/s, respectively.

The effect of the residence time at the various spike temperatures on the survival of nuclei is illustrated in Figure 12, showing the crystal fraction formed at the growth stage as a function of the spike time. The temperature protocol is similar to that in Figure 10, including nucleation at −30 °C for 200 s, growth at 0 °C for 1 s, and employing a supercritical nuclei-transfer heating rate of 2000 K/s. Spike temperatures and times were varied from 20 to 55 °C and from 0.01 to 1 s, respectively. In the case of applying a spike temperature of 55 °C (blue symbols), only a small number of the homogeneous nuclei survive on the path to the growth stage (see also Figure 11), and, consequently, the observed crystallinity is very small, close to zero, and independent on the spike-time when not exceeding 10 s. Annealing the sample at 55 °C longer than 10 s leads to crystallization via heterogeneous nucleation (see also the red symbols in the right plot of Figure 2b). When using lower spike temperatures of 35 and 20 °C (light- and dark-gray symbols, respectively), few crystals grew at the growth stage, however, with their number strongly increasing if the spike time exceeds about 0.2 s. Annealing the sample at 20 or 35 °C, which is close to the temperature range of the maximum crystal growth rate, for longer than 0.2 s allows crystal growth from homogeneous nuclei before transferring those nuclei back to the growth step at 0 °C. Annealing the sample containing homogeneous nuclei at a certain spike temperature too long will permit additional nuclei formation and eventually crystallization before reaching the growth stage. However, most important in the context of evaluation of the correct number of nuclei exposed to a certain temperature is the observation that there is no decrease with spike time, proving fast dissolution of nuclei, similar as it is true regarding the kinetics of melting of crystals.^81,82^ The results are consistent with a previous independent study.^80^

**Figure 13:**
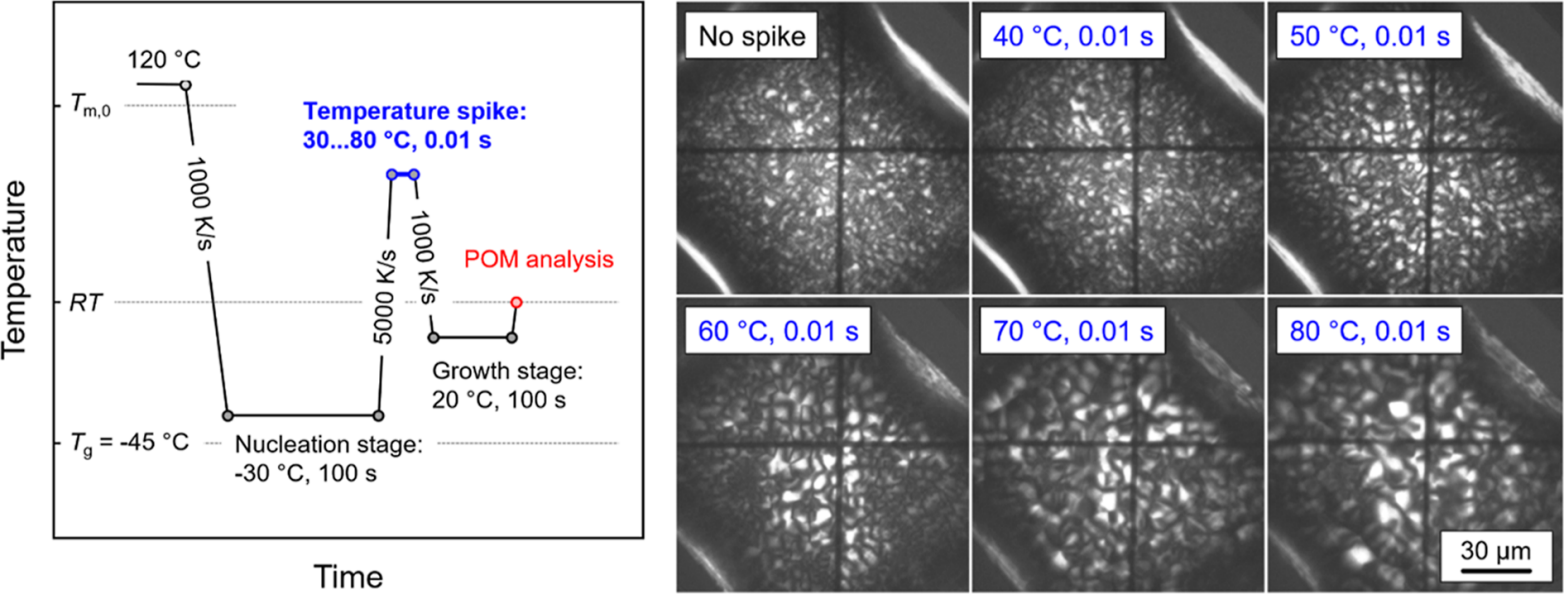
Temperature–time protocol for qualitative analysis of the thermal stability of homogeneous crystal nuclei of PBSA using a combination of FSC and POM (left) and resulting microstructures observed at room temperature as a function of the spike temperature. The scale bar holds for all images.

Additional, indirect information about the thermal stability of homogeneous crystal nuclei is collected by observation of POM images of FSC samples subjected to different spike temperatures, as illustrated in Figure 13. The left graph of Figure 13 shows the temperature–time protocol of the performed experiments, whereas the right images present POM microstructures of PBSA subjected to different spike temperatures on approaching the growth stage. Samples were melted at 120 °C and then quenched at 1000 K/s to the nucleation step at −30 °C, allowing nucleation for 100 s. Then, the samples were heated at 5000 K/s to different spike temperatures between 30 and 80 °C before permission of crystal growth at 20 °C for 100 s. Crystallization for 100 s is sufficient to fully crystallize the sample and stabilize the morphology until collection of the POM images at room temperature. After that, the samples were rapidly heated to room temperature for observation of their structures using POM. For comparison, a reference sample was prepared, without being exposed to a temperature-spike (see left top image), that is, the sample was directly transferred from the nucleation to the growth step. The reference shows a fine structure due to the rather high number of homogeneous nuclei formed at −30 °C, and with increasing spike temperature up to 80 °C, a continuous coarsening of the structure is observed, pointing to distinct changes of the nuclei number. The results of this experiment confirm the calorimetric observation that the thermal stability of the largest homogeneous nuclei formed at −30 °C within 100 s is 80–90 °C, that is, >100 K above the temperature of their formation. Note that the shift of a few K of the dissolution temperature of the largest nuclei in this experiment and the obtained data in Figure 11 (red circles) are probably caused by a temperature gradient due to a larger sample used for POM analyses.

## 4 Conclusions

The kinetics of homogeneous crystal nucleation and stability of nuclei of poly (butylene succinate-*ran*-butylene adipate) (PBSA) were analyzed using Tammann’s two-stage crystal nuclei development method. Regarding the overall crystallization kinetics, PBSA shows a slightly asymmetric temperature dependence with a minimum crystallization half-time of about 10 s near 20 °C. When compared to the PBS homopolymer, the crystallization rate of the specific PBSA investigated is about one order of magnitude lower, at least at temperatures higher than the crystallization-time minimum, when crystallization proceeds by heterogeneous nucleation. In this temperature range, crystallization of the melt yields the formation of spherulitically grown lamellae. In contrast, at high supercooling of the melt, a large number of nuclei develop, causing the formation of numerous birefringent objects including short and fine lamellae with a thickness of a few nanometers. Observation of such μm- and sub-μm sized birefringent objects may be related to coalescence of nanometer-sized crystals growing from a huge number of homogeneous nuclei, similar as in case of the butylene succinate homopolymer, or PLLA, but different from other polymers, like iPP, PA, or PBT.

Crystal nucleation and crystallization on cooling are suppressed if the melt is quenched to below *T*_g_ with rates higher than 100 and 20 K/s, respectively. Regarding the kinetics of homogeneous crystal nucleation, nuclei formation is fastest at about 0 °C, which is about 50 K higher than the glass transition temperature and begins after only a few seconds. Regarding analysis of the stability of nuclei, a spike-modified Tammann’s method was employed, revealing that the largest nuclei of the size distribution, generated at −30 °C within 100 s, are completely destroyed at 50–60 °C, that is, 80–90 K above their formation temperature, without any indication of an effect of time. This is confirmed by visual inspection of FSC samples using POM.

The study included an evaluation of the effect of the growth temperature on the critical transfer-heating rate to avoid nuclei-reorganization/-formation in Tammann’s method. The critical transfer-heating rate increases with the growth temperature at temperatures lower than the maximum of the crystallization rate. For PBSA, if the temperature difference between the nucleation and development temperature is less than 30 K, the critical nuclei-transfer heating rate is about 2000 K/s. A higher nuclei-transfer heating rate is required if the temperature difference exceeds 30 K, for compensation of the increasing growth rate when transferring the nuclei to a higher temperature. The latter result is considered important such that the specific design of Tammann’s experiment for analysis of the kinetics of homogeneous crystal nucleation requires the careful selection of the transfer-heating rate for each chosen combination of nucleation and development stages.
